# Breeding for Beneficial Microbial Communities Using Epigenomics

**DOI:** 10.3389/fmicb.2020.00937

**Published:** 2020-05-15

**Authors:** Kendall R. Corbin, Bridget Bolt, Carlos M. Rodríguez López

**Affiliations:** ^1^Environmental Epigenetics and Genetics Group, Department of Horticulture, College of Agriculture, Food and Environment, University of Kentucky, Lexington, KY, United States; ^2^Biosystems and Agricultural Engineering, College of Agriculture, Food and Environment, University of Kentucky, Lexington, KY, United States

**Keywords:** holobiont, crop breeding, sustainable agriculture, microbiome, epigenetics, epimutant populations

## Introduction

Traditionally, breeding programs have tapped into two main sources of diversity for the identification of alleles capable of conferring beneficial traits to the crop of interest: (1) The genetic diversity of landraces and wild relatives, and (2) the creation of novel alleles using random or directed mutation approaches.

The generation of mutant populations has been the base for many successful breeding programs, including barley, soybean, tomato, and wheat (Jankowicz-Cieslak et al., 2017). Genetic variability is used to develop novel agronomically beneficial traits but also to determine the putative function of non-characterized genes (reverse epigenetics), and to identify genomic locations responsible for traits of interest (forward epigenetics) in crops (Rodríguez López and Wilkinson, 2015). Genetic variation has been conventionally achieved through insertional mutagenesis (transfer DNA, transposons, and entrapment tagging) (Ram et al., 2019), chemical (i.e., ethyl methanesulfonate, EMS) or ionizing radiation (i.e., gamma ray) treatment (Jankowicz-Cieslak et al., 2017), and more recently through targeted gene editing approaches (TALEN, ZNF, and CRISPR/Cas9) (Wolter et al., 2019).

This approach has been pivotal in transforming food production systems but with an ever-changing environmental landscape and increasing global population, improvement rates fall short of providing food security (Mehrabi et al., 2018). Concerted research efforts have been made to address this pitfall. Advancements in recent years include marker-assisted selection for genes of interest (Karanjalker and Begane, 2016), the development of speed breeding methodologies to shorten generation time (Mehrabi et al., 2018), targeted breeding using directed approaches (as opposed to trial-by-error breeding strategies), reverse-breeding strategies to introduce genetic diversity (ancestral traits) back into commercial crops (Palmgren et al., 2015), and random chemical mutagenesis (Jankowicz-Cieslak et al., 2017). These progressive breeding programs have made strides in increasing crop quality and quantity, but it is increasingly recognized that they do not target all possible sources of phenotypic variability (Rodríguez López and Wilkinson, 2015).

## Epigenetic Mechanisms as a Source Variability for Crop Improvement

Epigenetic mechanisms regulate gene expression in response to plant development and environmental stimuli, ultimately affecting the plant's phenotype (Kumar, 2018). The field of applied epigenetics is a rapidly evolving area of research, stimulating new opportunities for the improvement of crop productivity. It is now widely accepted that epigenetic mechanisms have been the source of useful variability during crop varietal selection (Rodríguez López and Wilkinson, 2015; Crisp et al., 2016; Fortes and Gallusci, 2017; Gallusci et al., 2017). An early example of epigenetic breeding demonstrated the potential to improve crop performance and energy use efficiency (an important yield determinant) in a commercially valuable crop, rapeseed (*Brassica napus)*, through recurrent epigenetic selection of isogenic lines (Hauben et al., 2009).

The major epigenetic mechanisms mediating these effects include histone modifications, DNA methylation and small RNA molecules, which act in an interactive, and redundant fashion to affect gene expression (Rodríguez López and Wilkinson, 2015). DNA methylation involves the addition of a methyl group to the 5th carbon of cytosines (forming 5-methylcytosine) by a set of enzymes called DNA methyltransferases. Gene promoter methylation has been associated to transcriptional repression (Kass et al., 1997). Importantly, this classic promoter methylation–gene expression model does not seem to be universal (Anastasiadi et al., 2018). A more complex model has been suggested where the methylation of the promoter and the gene body exerts separate influences on gene expression (Wang et al., 2015). In general, a negative association has been found between gene body methylation (GbM) and gene expression (Anastasiadi et al., 2018; Magris et al., 2019). Nevertheless, GbM has also been linked to higher gene expression in certain gene subclasses (Dubin et al., 2015; Anastasiadi et al., 2018).

Exploiting the relationship between gene DNA methylation and expression through deliberate perturbation of DNA methylation via exogenous interventions, has been proposed as a fast method to generate variability for crop improvement (Rodríguez López and Wilkinson, 2015; Gallusci et al., 2017). This can be achieved by using methods that are analogous to those used in mutation breeding, application of chemical inhibitors of DNA methyltransferases, which causes stochastic genome-wide DNA demethylation (Geyer et al., 2011; Amoah et al., 2012; Browne et al., 2020) and so, generates new variants carrying epi-alleles (defined here, as any of a group of otherwise identical genes that differ in the extent of their methylation). The use of targeted epigenome editing techniques capable of altering DNA methylation or histone modifications in the genes of interest may also be employed (Vojta et al., 2016). The induction of such epialleles lead to changes in gene expression and phenotype. This strategy, similar to mutation breeding, can be used to generate novel, and valuable epigenetic variation for crop improvement (Amoah et al., 2012). Novel epialleles can be inherited, even over multiple rounds of sexual reproduction, (Amoah et al., 2012; Tricker et al., 2013a,b). More importantly, they can become fixed in hybrids, resulting in heritable molecular and physiological phenotypes (Wibowo et al., 2018) without the need for genetic modification.

## The Holobiont as a Potential Breeding Target

**Figure 1:**
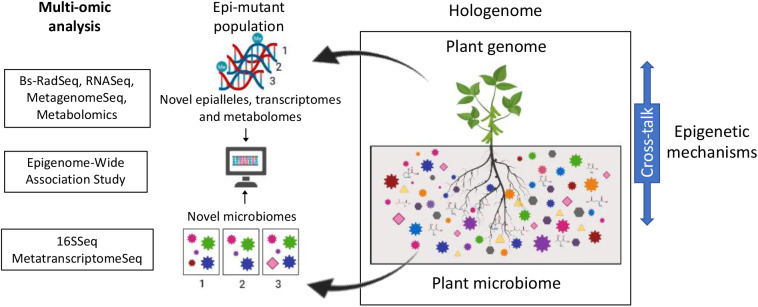
Proposed model for the identification of genes regulating plant-microbe interactions via the stochastic perturbation of DNA methylation patterns in plant populations. Exogenously induced DNA demethylation randomly generates novel epialleles in the plant population. These in turn alter the expression of genes which will alter the plant phenotype (i.e., microbiomes) of the plants carrying such novel alleles. Changes in DNA methylation in the plant population can be assessed using reduced representation approaches such as BsRADSeq. Changes in microbiome composition and functionality can be assessed using 16S sequencing and/or Meta-transcriptome analysis. Association between individual changes in DNA methylation (novel epialleles) and changes in phenotype (novel microbial community composition and functions) will be determined using epigenome wide association studies. Analysis of plant gene expression and metabolite production will be used as a validation step of the identified associations.

Thus far, breeding approaches consider the crop as a single species. However, in nature, plants do not exist as an entity, but cohabit with diverse microbes (collectively termed the plant microbiota). The assemblage of the host and the microbiota is referred to as the holobiont, while the term hologenome is used to indicate the entire set of genomes within the holobiont (Figure 1).

Microbial communities provide multiple benefits to their hosts, including better access to nutrients, enhanced growth, and improved tolerance to biotic and abiotic insult (Powell et al., 2015; Harman and Uphoff, 2019). The realization of the importance of microbiotas for crop health, has led to the development of prebiotic and probiotic cocktails intended to enhance the holobiont (Rodriguez et al., 2019). However, their effectiveness has been proven to be highly inconsistent (Ownley et al., 2003). This inconsistency has been attributed to different causes, including the host plant or pathogen genotype (Yang et al., 2018), agricultural practices (Schippers et al., 1990), and loss of activity due to mutation of the biocontrol strain (Duffy and Défago, 2000). These findings highlight the on-going need to better understand the host-microbiota interactions for crop improvement, and suggest non-specific additive cocktails are sub-optimal for general application (Rodriguez et al., 2019).

Interestingly, a handful of recent studies have shown that crop domestication and breeding have inadvertently altered the microbial communities of the target crops (Leff et al., 2017; Chaluvadi and Bennetzen, 2018), suggesting that microbiota composition is a trait that can be bred (Wissuwa et al., 2009). Unfortunately, very little research effort has been invested in understanding host-microbe interactions from a community perspective (Beilsmith et al., 2019). This makes the understanding of what makes a “good microbiota host” critical in the conceptualization of breeding programs aimed at improving productivity, quality and sustainability through the management of the holobiont (Wissuwa et al., 2009).

## Drivers of Soil Microbiome Composition

The below-ground microbiota is considered the richer and more functionally active of all the plant's compartments and is consequentially the most intensely studied (Rodriguez et al., 2019). The structural and functional diversity of the plant microbiota fluctuate in response to environmental and host pressures, creating a biological feedback loop (for an extensive review see Vorholt, 2012).

Although abiotic cues, such as soil physical and chemical characteristics, climate, and spatial features, have traditionally considered the main drivers of the plant microbiota composition (Weckert, 2016; Delgado-Baquerizo et al., 2020), it is now well-established that host factors such as genotype (Bulgarelli et al., 2015; Chaluvadi and Bennetzen, 2018), developmental stage (Sugiyama et al., 2014; Wagner et al., 2016) and plant organ (Wagner et al., 2016) contribute to the shaping and maintenance of the plant's microbial communities (Rodriguez et al., 2019).

## The Plant as a Driver of its Own Microbiota

The plant itself plays a key role in shaping the composition and relative abundance of microbial species in their rhizosphere through physical (e.g., root architecture) (Chaluvadi and Bennetzen, 2018; Saleem et al., 2018) and chemical mechanisms (i.e., exudation of small molecules that serve as growth substrates or signals for suitable microbial partners, and as antimicrobials or growth deterrents for other microbes) (Bais et al., 2006). Interestingly, the diversity of the microbial community sharply decreases with proximity to the plant (bulk soil>rhizosphere>endophytic compartment) (Rodriguez et al., 2019). This observed decline in diversity suggests that plants impose a strong selective pressure on their immediate surroundings (Bais et al., 2006).

Historically, the plant's genotype has been recognized as one of the key factors mediating this selectivity. The genotype impacts the microbial community to promote plant growth, improve abiotic stress tolerance, facilitate pathogen defense (Jones et al., 2019). However, Wibowo et al. (2018) showed that genetically identical plants, displaying distinct epigenomes differentially alter their microbiota. Symbionts have been shown to provide beneficial selectable variation to their hosts through the modification of the epigenetic profiles (Gilbert et al., 2010; Gómez-Díaz et al., 2012). Moreover, multiple studies have highlighted the importance of epigenetic mechanisms in regulating the cross-talk between the host and its associated microbiota (Figure 1; Gómez-Díaz et al., 2012; Cheeseman and Weitzman, 2015; Wang et al., 2016; Zhu et al., 2016; Kumar et al., 2018). Enhancing this genome-microbiome communication could be the target for future breeding programs.

## Epimutagenesis: a Tool to Identify Genes Regulating Host-Microbiome Interactions

Understanding what makes a plant a good host for its microbiota will be essential to harness the plant-microbiota complex for crop improvement. Identifying the genes that enable plants to regulate the assembly of a beneficial root microbiota is paramount for future breeding programs aimed at a sustainably improving productivity and produce quality of produce. Although, very little is known about the molecular mechanisms regulating the assembly of plant microbiota, multiple studies have pointed at the importance of epigenetic mechanisms regulating the cross-talk between the host and its associated microbiota. We propose that capitalizing on the availability of epimutant populations as a platform for the identification of loci involved in the regulation of plant microbiota assemblies using epigenome wide association studies (EWAS) (Flanagan, 2015; Birney et al., 2016; Jullian Fabres et al., 2017; Figure 1). The rationale behind the use of an EWAS approach resides on the stochastic nature of the epimutations induced by the application of exogenous demethylating agents. This approach would generate a unique set of epialleles in each plant within the epimutant population, which could partially alter the plant's ability to direct the assembly of its microbiota. Identifying the genes regulating host/microbe interactions will provide with valuable targets for breeding aiming at producing crops capable of assembling healthier microbiotas. This in turn, has the potential to aid global efforts in addressing the challenge of feeding a growing population via the development of socially and environmentally responsible agricultural approaches (Mehrabi et al., 2018).

## Author Contributions

All authors listed have made a substantial, direct and intellectual contribution to the work, and approved it for publication.

## Conflict of Interest

The authors declare that the research was conducted in the absence of any commercial or financial relationships that could be construed as a potential conflict of interest.
